# A Novel Delayed Phase Inversion Strategy Enables Green PVDF Membranes for Membrane Distillation

**DOI:** 10.3390/membranes14110241

**Published:** 2024-11-15

**Authors:** Wenbin Sun, Longbo Xia, Ping Luo, Dong Zou

**Affiliations:** 1 School of Environmental Science and Engineering, Nanjing Tech University, Nanjing 211816, China; sunwenbin@njtech.edu.cn (W.S.); 202261202058@njtech.edu.cn (L.X.); 2 State Key Laboratory of Polymer Materials Engineering, Sichuan University, Chengdu 610065, China

**Keywords:** PVDF membrane, delayed phase inversion, green fabrication, membrane distillation

## Abstract

Polyvinylidene fluoride (PVDF) membranes are extensively utilized in membrane distillation (MD) for water treatment. However, traditional methods easily form asymmetrical membranes with dense skin layers that are detrimental to membrane flux. Herein, an eco-friendly PVDF membrane was fabricated by utilizing a delayed phase separation process without using any pore-forming agents. In addition, methyl-5-(dimethylamino)-2-methyl-5-oxopentanoate (PolarClean) was used as a green solvent without posing risks to humans and the environment. It was demonstrated that the PVDF concentration is crucial in influencing the microstructures and performance of the resulting membranes. As the PVDF concentration increased, the morphology changed significantly, resulting in a reduction of pore size. When feeding the device with NaCl solution at a concentration of 35 g/L, the MD water vapor flux reached 18.49 kg·m^−2^·h^−1^, while maintaining a salt rejection of over 99.97% during the continuous operation for 24 h. This work presented a method for producing green PVDF membranes via delayed phase inversion with satisfactory water vapor flux and salt rejection, highlighting their prospect for effective applications in MD for water treatment.

## 1. Introduction

Seawater treatment has advanced rapidly due to increasing water shortages [1,2,3]. Membrane distillation (MD) is widely used for desalination because of its mild operating conditions and energy efficiency [4,5,6]. Among them, polyvinylidene fluoride (PVDF) is widely utilized in the preparation of membranes for seawater treatment, owing to its exceptional chemical degradation resistance, mechanical strength, thermal stability, and contamination resistance [7]. Non-solvent induced phase separation (NIPS) is a typical method to fabricate PVDF membranes [8,9,10]. However, the NIPS process depends on many factors, including the type of solvent, components of the coagulation bath, and temperature [11]. Among them, solvent is the main component of the casting solution that accounts for about 70~90%. Polar aprotic solvents, like N-methyl pyrrolidone (NMP) [12], N-N-dimethylacetamide (DMAc) [13], and N-N-dimethylformamide (DMF) [14] are extensively utilized due to their chemical affinity with polymers. However, these solvents pose a serious threat to health and the surroundings. For example, NMP can cause irritation to the skin and respiratory tract. DMF is carcinogenic, and DMAc has some reproductive toxicity. These solvents are not only limited to the preparation of the casting solution but also remain in the wastewater after membrane formation. According to statistics, more than 50 billion liters of sewage are poured annually [15]. Therefore, the adoption of less toxic or environmentally friendly solvents is essential for sustainable membrane technology.

Methyl-5-(dimethylamino)-2-methyl-5-oxopentanoate (PolarClean) is a recently discovered eco-friendly green solvent [16]. Polarclean is a clear, slightly yellow liquid with boiling and freezing points (at 1013 hPa) 278−282 °C and –60 °C, respectively; the flash point (closed cup) is 144−146 °C. The water solubility of Polarclean is higher than 490 g dm^−3^ at 24 °C [17]. It has the advantages of low toxicity, good biodegradation, and environmental protection, which reduce harmful emissions [18]. In addition, PolarClean is completely miscible with water and has a certain solubility for polymers, which can be utilized as a solvent to prepare PVDF membranes via the NIPS method [19,20]. However, it is generally recognized that the rapid transfer of solvents and non-solvents during the inversion phase can result in a dense membrane structure, which is not conducive to vapor transport in the MD process [21]. Currently, studies on methods to construct porous surfaces are mainly focused on adding pore-forming agents [22,23,24], changing bath components [25], and surface modification [26,27], while fewer studies have been conducted on changing the membrane structure through the preparation process [28]. Tian et al. [21] proposed a co-casting method that constructed a polymer isolation layer on the PVDF membranes to limit the exchange of solvents and non-solvents to prepare hydrophobic microporous PVDF membranes. Through this method, the PVDF membranes obtained a porosity of about 45% and a water flux of 25 kg·m^−2^·h^−1^. Marcello et al. [29] investigated how the support structure and materials influenced membrane morphology and MD performance. The outcomes showed that the PVDF membranes prepared by non-woven fabrics (NWFs) had a flux of over 60 kg·m^−2^·h^−2^. Wu et al. [30] further investigated the role of the NWFs on the membrane microstructure and its performance during the phase inversion process from a kinetic point of view. They analyzed how NWFs influence the membrane properties and structure during phase inversion from a dynamic perspective and found that the structure embedded in NWFs provided extremely high mechanical strength (~50 MPa).

In this work, PolarClean served as a solvent to realize a green process for membrane fabrication. In addition, the NWFs were employed to act as a physical obstacle to delay the phase inversion process of PolarClean and water during membrane formation. The impacts of PVDF concentrations on wettability, pore size, roughness, morphology, and porosity were studied. Then, the optimized PVDF membranes were prepared and placed in the MD system to test the membrane performance, including the salt rejection and water vapor flux of simulated seawater. The work provides an interesting and eco-friendly approach to fabricating PVDF membranes for MD applications.

## 2. Experimental

### 2.1. Materials

PVDF power (Solef 1015, Molecular weight: ~573,000) was obtained from Solvay Co., Ltd. (Shanghai, China). Sodium chloride (NaCl, ≥99.5%) was obtained from Shanghai Aladdin Biochemical Technology Co., Ltd. (Shanghai, China). PolarClean was obtained from Solvay Novecare (Cranbury, NJ, USA). Deionized water (DI water, 13–17.5 MΩ cm) was supplied from an ultrapure water machine (Q2-10T, Nanjing Yuheng Instrument Equipment Co., Ltd., China).

### 2.2. PVDF Membrane Fabrication

**Figure 1:**
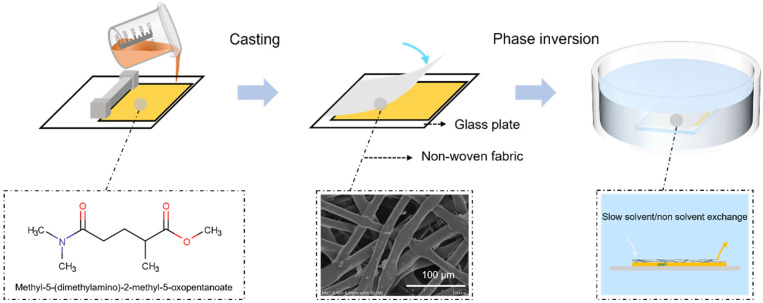
Preparation diagram for PVDF membrane by delayed phase inversion.

Figure 1 shows the specific process for preparing the PVDF membranes. First, the required mass of the PVDF powders that were used to prepare PVDF membranes at different concentrations was weighed. Then, the powders were placed in a three-neck flask and dissolved in PolarClean via mechanical stirring at 140 °C for 2 h to obtain homogeneous solutions. The solutions were maintained at 140 °C for 3 h to ensure that all bubbles were removed. Next, the casting solution was poured onto a clean glass plate before a membrane layer with a thickness of 300 μm was scraped out. Subsequently, the NWFs were instantly covered on the membrane layer. Finally, the membrane was placed in water (20 °C) for 24 h and dried for 6 h after the solvent from the matrix of the PVDF membranes was removed. The chemical formula of Polarclean and the surface structure of the NWFs are displayed in Figure 1.

### 2.3. Characterization

The scanning electron microscope (FE-SEM S-4800, Hitachi, Tokyo, Japan) was utilized to examine the surface structures of the membranes at an acceleration voltage ranging from 5 to 7 kV. The atomic force microscope (AFM, PARK XE-100, Suwon, Korea) was used to measure the surface roughness on a specified 25 μm^2^ zone and then computed using the XEI5.0.1.Build5 analysis software. The hydrophobic nature of the membrane, as indicated by the water contact angle, was evaluated using the Dataphysios OCA25 (Filderstadt, Germany) by depositing 3 μL of water, and measurements were taken at four distinct regions. The mean pore size and pore distribution of the PVDF membranes were determined utilizing the bubble pressure method with a PMI ipore 1500 (Ithaca, NY, USA), ensuring complete wettability of the membrane with Galwick (surface tension of 15.9 dyn/cm). Nitrogen was used for pore opening, and the pore size (r) was computed according to Equation (1).

(1) $$∆P=\frac{2\sigma cos\theta }{r}$$

where Δ*P* denotes the additional pressure difference (MPa), *σ* stands for the liquid surface tension (N/m), and *θ* represents the water contact angle (°). The membrane’s total porosity was computed using the Equation (2):

(2) $$\varepsilon =1-\frac{M_{P}/\rho_{P}}{w\times l\times t}$$

In this equation, *M_P_* stands for the mass of the PVDF material (g), and *ρ_P_* stands for the density of the PVDF (1.75 g/cm^3^), while *t*, *l,* and *w* denote the thickness (cm), length (cm), and width (cm) of the membranes, respectively.

### 2.4. Membrane Distillation Performance

**Figure 2:**
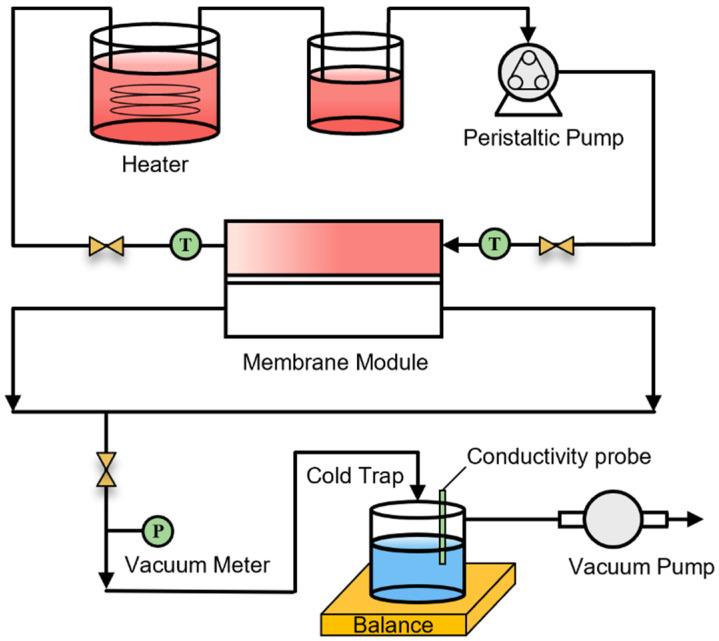
Schematic diagram of vacuum membrane distillation.

The desalination performance of the PVDF membranes was manifested through a vacuum membrane distillation (VMD) setup. The process of the VMD experimental device is shown in Figure 2. The concentration of the NaCl solution was 35 g/L, and the temperature of the NaCl solution was maintained between 50~70 °C. The DI water was put into the feed side every 2 h during the operation to keep a constant concentration. The circulation volume was controlled to be 600 mL. The feed solution was injected into the membrane through a peristaltic pump, and the flow rate was controlled at 200 mL/min. The effective area of the PVDF membrane in the module was 14.51 cm^2^ (diameter of 43 mm). The condensate was controlled by a chiller at a temperature of ±0.5 °C. The vacuum pressure was kept above −0.965 MPa during the test. The conductivity and mass of the permeate were monitored every half hour. The salt rejection and water vapor flux were calculated using Equations (3) and (4), respectively.

(3) $$R=\left(1-\frac{C_{P}}{C_{F}}\right)\times 100\%$$

where *C_P_* and *C_F_* stand for the salt concentration of the permeate and feed solution, respectively.

(4) $$J=\frac{∆m}{A\times t}$$

where Δ*m* (kg) stands for the mass of the condensate water within a fixed time loop (*t*, h), and *A* represents the effective area of the membrane (m^2^).

## 3. Results and Discussion

**Figure 3:**
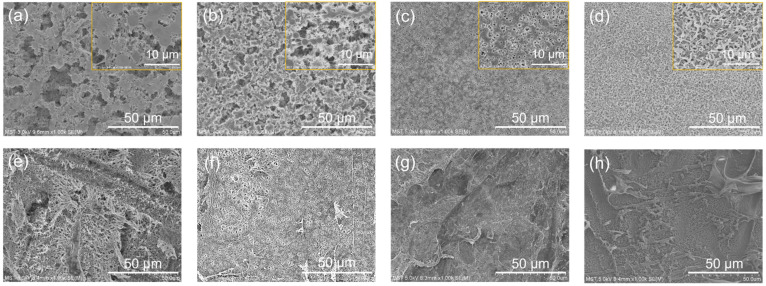
Effects of different PVDF concentrations on the morphologies of PVDF membranes. (**a**,**e**) 15 wt%, (**b**,**f**) 20 wt%, (**c**,**g**) 25 wt%, and (**d**,**h**) 30 wt%. (**a**–**d**) refer to the top surface, and (**e**–**h**) refer to the bottom surfaces. The yellow box in the upper right corner is the magnified image of the membrane top surface. Note that the bottom was observed after removing the NWFs.

Figure 3 displays the SEM results of the PVDF membranes at distinct concentrations. The surface is the side close to the glass plate, and the bottom is the side of the NWFs that sticks to the membrane. Compared with the traditional method, the delayed phased inversion can change the surface morphology and overall structure of the membrane. In the conventional method, the membrane layer came into direct contact with the water when the cast membrane was placed in the water [31]. The solvent (Polarclean) in the matrix of the membranes could exchange with water rapidly, resulting in the formation of a dense layer on the surface of the membrane [32]. However, the delayed phase inversion process in this work was different. In the membrane formation process, the NWFs were stuck to the nascent membrane surface. After being placed in the water, the NWFs acted as a physical barrier, slowing down the transfer rate between the solvent and water [33]. A porous surface was then produced. From Figure 3a–d, we can note that the delayed phase inversion method could produce porous PVDF membrane surfaces regardless of the PVDF concentrations. However, there is a slight difference in the microstructure of the PVDF membranes at distinct concentrations. When the concentration of PVDF ranged from 15 wt% to 20 wt%, the interconnected flower-like spherical particles appeared on the membrane surface. This was because the side in contact with the glass plate was isolated from the bath water when the membrane was soaked in a water coagulation bath. Therefore, the PVDF membrane obtained a long phase inversion time, resulting in the formation of oversized particles [34]. When the concentrations of PVDF were between 25 wt% and 30 wt%, the surface gradually tended to be intact, and the pore size became smaller. This is due to the fact that the growth of PVDF particles in a fixed volume was limited with the increase of polymer concentration, thus achieving a uniform smaller size. In addition, the bottom membrane surfaces are shown in Figure 3e,f. It can be seen that the formation of the microstructure of filamentous fibers was the imprint of the fiber texture from the NWFs. The fibrous structure of the NWFs further altered the diffusion behavior of water when it prevented the water from making direct contact with the nascent membrane. Additionally, the viscosity of the casting solution increased as the PVDF concentration rose, leading to the size coarsening of the fiber structures.

**Figure 4:**
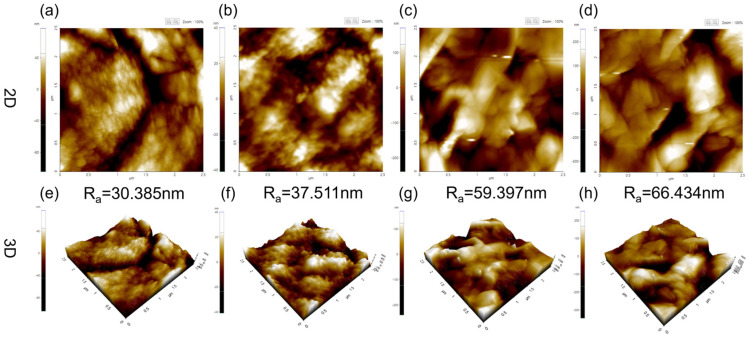
Surface roughness of PVDF membranes prepared at distinct polymer concentrations. (**a**,**e**) 15 wt%, (**b**,**f**) 20 wt%, (**c**,**g**) 25 wt%, and (**d**,**h**) 30 wt%.

Figure 4 shows the AFM results for various concentrations of PVDF membranes. We can see that when the PVDF concentration was 15 wt% and 20 wt%, the average roughness *(R_a_*) of the membranes was 30.38 nm and 37.51 nm, respectively. It is also interesting to point out that there were large pore defects on the membrane surface from the AFM characterization. Moreover, the mean roughness of the PVDF membranes changed from 59.39 nm to 66.43 nm as the concentration of the PVDF elevated from 25 wt% to 30 wt%. The increase in the average roughness can be ascribed to the diminished size of the PVDF particles on the membrane surface, which formed a rougher surface morphology [35]. The average roughness depended on the degree of surface roughness and the microstructure, while the surface of a single truncated sphere was relatively flatter.

**Figure 5:**
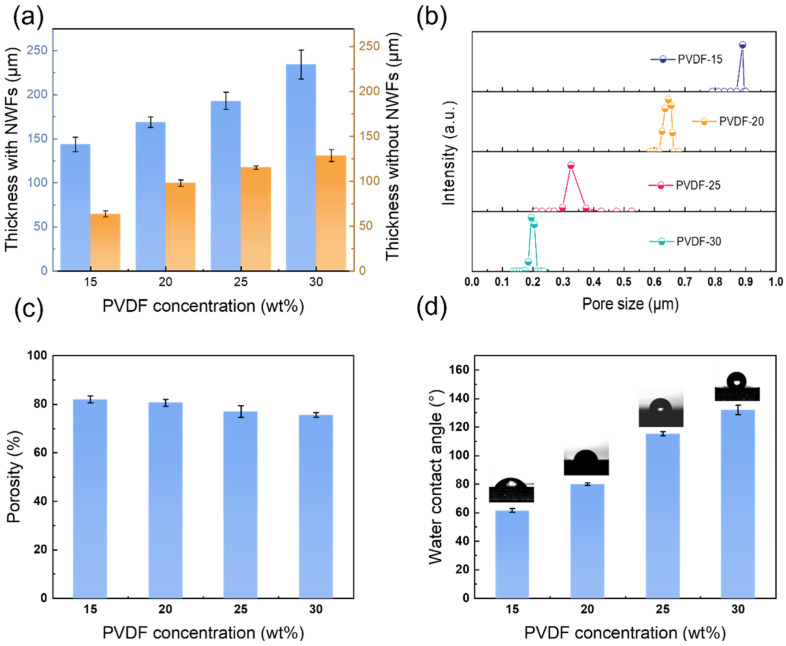
Properties of the PVDF membranes at various PVDF concentrations. (**a**) Membrane thickness, (**b**) pore size distribution, (**c**) porosity, and (**d**) water contact angle. Note that PVDF-15 indicates that the PVDF concentrations are 15 wt% and so forth.

Figure 5 displays the properties of the PVDF membranes at distinct polymer concentrations. Figure 5a shows the membrane thickness with or without using NWFs as supports. It can be found that the membrane thickness increased as the PVDF concentration (15~30 wt%) rose. The membrane thicknesses using NWFs were 143.6, 169.0, 193.1, and 234.3 μm, while the membrane thicknesses after removing the NWFs were only 63.6, 98.0, 115.3, and 128.6 μm, respectively. The casting solution became more viscous as the PVDF concentration increased, making it more difficult to penetrate into the pore structures of the NWFs. This could result in a higher membrane thickness. Figure 5b displays the pore size of PVDF membranes at distinct concentrations. It demonstrated that the pore size gradually became smaller with the rise of PVDF concentration. When the concentration of PVDF was 15 wt% and 20 wt%, the overall pore sizes were larger than 0.89 μm and 0.64 μm. That was because the high PVDF concentration in the cast membrane could decrease the porosity and thus lead to the formation of many small pores [36]. While the concentrations of the PVDF were 25 wt% and 30 wt%, the membrane integrity was satisfactory, with pore sizes of 0.35 μm and 0.19 μm, respectively. It was attributed to the smaller particle sizes (see Figure 3c,d). Figure 5c shows the porosity of the PVDF membrane. Apparently, the delayed phase inversion method maintained a high overall porosity of the PVDF membrane. As the PVDF concentration increased from 15 wt% to 30 wt% (with increments of 5 wt%), the corresponding porosity values were 82%, 80%, 77%, and 75%, respectively. Figure 5d shows the water contact angle that reflected the hydrophobic properties. It can be observed that the water contact angle rose from 61.6°, 80°, 115.4° to 132.1° as the concentration of PVDF was elevated from 15 wt% to 30 wt%, respectively. The variations in the contact angle with water were positively correlated with the roughness of the PVDF membranes. High membrane roughness could lead to increased hydrophobic properties and a higher water contact angle [37].

**Figure 6:**
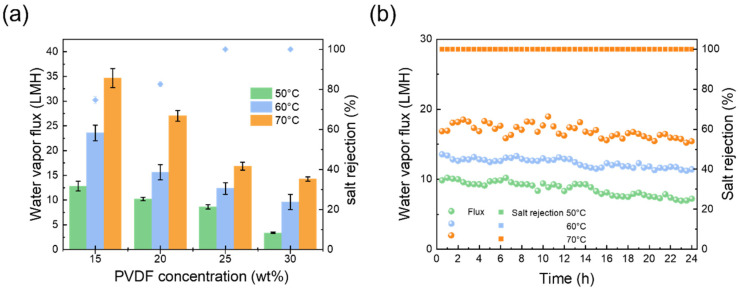
(**a**) MD performance of PVDF membranes at various PVDF concentrations, and (**b**) membranes with a PVDF concentration of 25 wt% underwent long-term performance testing.

Figure 6 shows the effect of the different concentrations of PVDF membranes on membrane distillation performance. We can note that the vapor fluxes were higher when the concentration of PVDF was 15 wt% and 20 wt%. However, the salt rejection was only 74.68% and 82.57%, respectively. This was caused by the non-uniformity of the pore size on the membrane surface, which did not completely reject the saline water. Additionally, this finding suggests that the vacuum MD system is not suitable for PVDF membranes with pore sizes of 0.89 μm and 0.64 μm [38]. In contrast, during the entire process, the salt rejection remained over 99.9% when the concentration of PVDF was 25 wt% and 30 wt%, respectively. Specifically, the average vapor fluxes at 50, 60, and 70 °C were 8.6, 12.4, and 16.9 kg·m^−2^·h^−1^ (25 wt%) and 3.4, 9.7, and 14.2 kg·m^−2^·h^−1^ (30 wt%), respectively. This was because the PVDF membrane with a concentration of 25 wt% exhibited higher porosity and thinner thickness, thereby obtaining a lower transferring resistance. Therefore, the optimized PVDF concentration was selected as 25 wt%, and the resulting PVDF membranes were employed for a 24 h long-term MD test (Figure 6b). The fluxes varied from 7.22 to 10.21 kg·m^−2^·h^−1^ at 50 °C, and from 11.40 to 13.55 kg·m^−2^·h^−1^ at 60 °C. At 70 °C, the vapor flux decreased slightly from 18.49 kg·m^−2^·h^−1^ to 15.21 kg·m^−2^·h^−1^. Throughout the process, the salt rejection consistently exceeded 99.97%, indicating a high MD separation performance.

## 4. Conclusions

The delayed phase inversion method effectively slowed down the transfer between solvents and non-solvents. This method successfully fabricated a hydrophobic and porous PVDF membrane using green solvents. Specifically, NWFs hindered the diffusion behavior of water and solvents, which delayed the occurrence of phase inversion. This method induced a porous surface with interconnected spherical particles, and the total porosity of the membranes was relatively higher than ~79 ± 3%. By increasing the PVDF concentration from 15 wt% to 30 wt% in 5 wt% increments, the membrane thickness elevated from 63.6 μm to 128.6 μm, respectively, and the pore size reduced from 0.89 μm to 0.19 μm, respectively. At the optimized condition (a PVDF concentration of 25 wt%), the resulting membranes were applied well to MD for seawater desalination. At 70 °C, the water vapor flux of these membranes ranges from 18.49 to 15.21 kg·m^−2^·h^−1^. The salt rejection was stably maintained above 99.97% in the MD test for 24 h.

Finally, it is important to mention the following fact. Different from the conventional NMP solvent, PolarClean must be heated to 130–140 °C to dissolve the PVDF particles, which could increase the fabrication cost. In the future, more green solvents that could dissolve PVDF powders at room temperature should be considered.

## Data Availability

The original contributions presented in this study are included in the article. Further inquiries can be directed to the corresponding authors.
